# The correlation of osteoporosis to clinical features: a study of 4382 Female Cases of a Hospital Cohort with musculoskeletal symptoms in Southwest China

**DOI:** 10.1186/1471-2474-11-183

**Published:** 2010-08-16

**Authors:** Shasha Li, Hongchen He, Mingfu Ding, Chengqi He

**Affiliations:** 1 Department of Rehabilitation Medicine, West China Hospital, Sichuan University, Chengdu, Sichuan Province, China; 2 Province Key Laboratory of Rehabilitation Medicine, Chengdu, Sichuan Province, China

## Abstract

**Background:**

By analyzing the clinical features and risk factors in female patients with musculoskeletal symptoms of Southwest China, this report presents the initial analysis of characteristics in this region and compared with international evaluative criteria.

**Methods:**

Diagnosis of osteoporosis (OP) was made in female hospital patients age ≥ 18 years admitted from January 1998 to December 2008 according to WHO definition. Case data were analyzed by symptoms, age, disease course and risk factors to reveal correlation with diagnosis of OP. Logistic regression was used to identify the risks of osteoporosis.

**Results:**

A total of 4382 patients were included in the analysis of the baseline characteristics, among which 1455 in the OP group and 2927 in the non-OP group. The morbidity of OP is significantly increased in females' ≥ 50 years. Both groups had symptoms related to pain and numbness; no significant difference was found in reported upper and lower back pain, or leg pain between two groups (*p *> 0.05). Neck, shoulder and arm pain, leg and arm numbness were more common in the non-osteoporosis group (p < 0.05, OR < 1, and upper limit of 95% CI of OR < 1). Hypertension, diabetes, hyperostosis were major risk factors for the patients with OP. The most common lifestyle-related risk factors for osteoporosis were smoking, body mass index, lack of physical activity and menopause.

**Conclusions:**

The present study offers the first reference data of the relationship between epidemiologic distribution of osteoporosis and associated factors in adults Chinese women. These findings provide a theoretical basis for its prevention and treatment in developing country.

## Background

Osteoporosis (OP) is a general skeletal disease predominant in aged adults, particularly in postmenopausal women, characterized by osteopenia and degeneration of bone microstructure, leading to increasing bone brittleness and tendency for bone fracture [1]. According to Cochrane systemic reviews, bisphosphonates including alendronate, etidronate and risedronate are clinically important and statistically significant reductions in vertebral fractures for secondary prevention [2-4]. While no standard therapeutic regimen for primary prevention is recommended. Early risk factors identification and early intervention in high-risk groups is important to prevent osteoporosis and bone fracture. The clinical symptoms of osteoporosis include pain, decreased body height, dowager's hump, bone fracture and respiratory impairment. The optional method to diagnose osteoporosis is by measuring bone mineral density (BMD) with dual-energy x-ray absorptiometry (DEXA) at the hip and lumbar spine. DEXA is recommended by the World Health Organization (WHO) in 1994 as the gold standard to measure BMD to diagnose asymptomatic osteoporosis [1]. According to diagnostic criteria by BMD, 0.6% of young women (those 20-49 years) have osteoporosis and 16% have osteopenia, while in white women ≥ 75 years, 38% have osteoporosis and 94% have osteopenia [5]. The optimal method is the use of clinical risk factors for fractures together with DXA to diagnosis and evaluation of patients with osteoporosis in 2002 [6] At present, The FRAX^® ^tool has been developed by WHO to evaluate fracture risk of patients [7]. It is based on individual patient models that integrate the risks associated with clinical risk factors as well as bone mineral density (BMD) at the femoral neck. However, these methods were not widely applied in developing countries due to lack certain information channel.

Osteoporosis is a recognized major public health problem in both developed and developing countries. In China, about 83.9 million people were diagnosed with osteoporosis in 1997 [8]. As the age span has increased, osteoporosis has become the fourth most common disease in aged adults. Due to the high degree of morbidity and mortality associated with fracture, prevention of such events is imperative. Because the number of women at risk for osteoporosis is expected to rise dramatically with the aging world population, effective means to predict more accurately the prevalence of osteoporosis and related risk factors in communities is critical [9]. The prevalence in China remains unclear.

Southwest China has a larger population (87 millions) and has a socioeconomic status that is below the Chinese average. No studies of clinical data of osteoporosis in this region have been previously reported. Thus, studies characterizing the epidemiology of osteoporosis and associated factors in Chinese females are required. To begin to characterize clinical osteoporosis in this population, we performed a retrospective cross-sectional survey of osteoporosis, including demographic and social information, musculoskeletal symptoms, medical complications, history of bone fracture, and disease course and lifestyle factors in female subjects using 10 years of hospital data.

## Methods

### Patients

This study was approved by the institutional ethics committee at Sichuan University and written informed consents were obtained from all subjects prior to examination. Research carried out in compliance with the Helsinki Declaration [10]. This retrospective analysis of consecutively collected data was performed in the West China Hospital of Sichuan University. The total of 4382 adult women age ≥ 18 years hospitalized in the Department of Rehabilitation Medicine and the Orthopedics Department for pain or numbness in bones or joints from January 1998 to December 2008 were consecutively registered. All these patients were with the chief complaint of osteoarthralgia and at least one of these clinical symptoms: 1) neck and shoulder pain; 2) lumbar and back pain; or 3) extremity pain or numbness. Those who with continued cessation of menstruation for more than 12 months were recognized as postmenopausal women, and the rest were categorized as premenopausal or perimenopausal women. Of these 4382 patients, 3399 were postmenopausal, the mean age at menopause was 43.5 ± 4.15 years (mean ± SD; range, 39 - 58 years) with a mean duration after menopause of 13.1 months. The data from all cases were recorded consecutively from 1998 and all data were submitted to the OP Research Center and the diagnoses were confirmed according the findings of DEXA. The diagnosis of OP in this study was established using WHO criteria,^1 ^so that osteoporosis was defined as a T-score of less than -2.5 SD. Osteopenia denoted a T-score of -1 to -2.5 SD. Exclusive criteria included: 1) diseases which interfere with bone or calcium metabolism; 2) hepatic or renal inadequacy; 3) taking medicines which could interfere with bone or calcium metabolism (such as estrogen, calcitionin, etc.); 4) limited mobility such that the patient could not position for the bone density test; and 5) secondary (i. e. steroids, plasmocytoma, or renal) osteoporosis.

### Questionnaires

Osteoporosis registration forms were filled out for all the female patients by graduate students majoring in osteoporosis research who had received standard training. The graduate students collected the following information: demographic information, medical history such as hypertension, diabetes mellitus, and hyperostosis, clinical features, physical examination, lifestyle factors, BMD value (T-Score), complications such as bone fracture. Demographic information including age, menstrual history, education level, body mass index (BMI) and home address. Education level was categorized in three groups, by number of years of schooling: low level (< 6 years), medium level (6-9 years), and high level (> 9 years). Patients were interviewed about their level of physical activity classified as never, occasionally (two to five times a week, more than half an hour every time), or frequently (more than five times a week, more than half an hour every time). All the information was confirmed by the patients or a reliable relative.

Detailed clinical manifestation included a history of complaint, the type of symptoms (neck and shoulder pain; lumbar and back pain; or extremity pain or numbness and number of painful or stiff joints), history of fracture (hip, spine, wrist, rib, or any site of bone), medical comorbidities (hypertension, diabetes mellitus, and hyperostosis), the duration of symptoms before treatment, the duration of osteoporosis and DEXA findings. The database was selectively examined periodically to ensure the accuracy of the extracted information and input data.

### Bone Mineral Assessment

Bone mineral density at site of lumbar vertebrae (L2-L4) was measured using the dual emission X-ray absorptiometry scanner (FR DMS Corp, Challenger, France) at the supine lumbar spine, including the posteroanterior (PA) vertebrae L1-L4, followed by a paired PA/lateral spine scan of the vertebral bodies of L2-L4; at the left hip, including the femoral neck and total hip. BMD was expressed in g/cm^2^. The coefficient of variation (CV) of the technique at our institution was 0.4%, using a phantom measured everyday during the 10-year period of the current prospective study. We used Asian spine reference population for young women based on the age range 20-40 years. T-Scores were calculated using the standard formula as follows: T-score = BMD of participant mean-mean BMD of reference population/SD of BMD of reference population. Cut-off values to categorize individuals as having low bone mass (osteopenia) or osteoporosis utilized the WHO criteria [1].

Measurements were made by one of two trained and qualified technicians, and all scans were analyzed by a single researcher using standardized procedures as outlined in the DMS User Manual [11].

Consecutively registered patients to establish an osteoporosis database and retrospective observed the clinical features of osteoporosis and analyzed the factors that may affect its prognosis.

### Data Processing and Statistical Analysis

In the univariate analysis, mean values of age, BMI, T values and duration of osteoporosis were analyzed as continuous variables, while frequency of physical activity, clinical symptoms, history of bone fracture, complications, smoking and calcium supplements were analyzed as categorical variables. To examine the baseline differences of participants by group, we calculated each group's means and percentages and performed T-test or chi-square test to examine baseline differences by group. According to the WHO's definition [1], women patients were classified as osteoporotic if they had a T score of -2.5. We divided the patients into two groups according to the above criteria of osteoporosis. Incidence rates of pain in the neck, shoulder, lower back, upper back, leg or arm and numbness in one or more extremities in the osteoporosis group and non-osteoporosis group were compared. The results for the whole cohort and subgroups were compared. The choice of subgroups was based on the phase of menopause at baseline. The data were analyzed in subgroups for osteoporosis group according to the following categories: premenopausal, perimenopausal and postmenopausal women.

The correlation of clinical symptoms, average age, average course of disease and complications with bone densities of lumbar vertebrae was also analyzed in osteoporosis patients.

Statistical analysis was conducted with SPSS 13.0 software (SPSS, Inc., Chicago, IL). Continuous variables were expressed as mean ± standard deviation (SD); those in a category were expressed as a frequency (percentage). T-test was applied to examine the statistical differences between the two groups. Numerical data were compared by chi-square. The normal distribution of variables was confirmed by calculating skew and kurtosis before applying standard tests. One-way analysis of variance (ANOVA) was applied when comparing differences of groups with a post-hoc test using Tukey's method. One-way ANOVA was used to compare continuous variables between multiple groups and Chi-squared tests to compare proportions. Factors that may influence prognosis of osteoporosis including age, course of disease, lifestyle factors, clinical symptoms, and complications were fitted into a logistic regression model for multi-variable analysis. A P value of < 0.05 was considered statistically significant. Odds ratio (OR) value and 95% Confidence interval were calculated and tested for significance.

## Results

### Demographic Variables

All participants completed a structured questionnaire. Data were collected from the completed checklists and the patients' electronic medical records. These data sources were reviewed and extracted for patient demographics including current age and age at menarche and menopause, comorbidity conditions, medication history, and life style.

A total of 4382 patients were included in the analysis of the baseline characteristics, among which 1455 in the OP group and 2927 in the non-OP group. Table 1 shows demographic baseline of the two groups taking part in this study. Overall, two groups showed similar demographic characteristics, except that patients in osteoporosis group were older (t = 2.355, p = 0.014) and had a lower height (t = 2.054, p = 0.038) than those in non- osteoporosis group.

**Table 1:** Demographic baseline of OP group and non-OP group

	Osteoporosis group	Non-Osteoporosis Group
	(n = 1455)	(n = 2927)
**Current age (yrs)**	66.5 ± 8.5*	62.4 ± 7.8
**Age at menarche (yrs)**	12.4 ± 3.5	12.5 ± 4.1
**Age at menopause (yrs)**	43.3 ± 5.3	45.2 ± 4.7
**Current height (m)**	1.52 ± 0.21*	1.59 ± 0.25
**Current weight (kg)**	54.5 ± 5.6	53.6 ± 4.

* Osteoporosis vs. non-osteoporosis, t-test, P < 0.05

### Results from Bmd Measurements

According to osteoporosis is defined as a T-score of less than -2.5 SD and osteopenia denotes a T-score of -1 to -2.5 SD, Table 2 shows the results of the DEXA measurements and the proportion of patients who had osteoporosis, osteopenia and normal BMD at same skeletal sites. Of these 4382 women, 33.20% (1455 cases) had osteoporosis. The corresponding percentages for osteopenia were 20.45% (896 cases) and 46.35% (2031 cases) were with normal BMD.

**Table 2:** Age-stratified analyses of case-distributions and BMD measurements

Age (years)	Osteoporosis (n = 1445)		T value (mean ± SD)	Osteopenia (n = 896)		T value (mean ± SD)	Normal BMD (n = 2031)		T value (mean ± SD)
						
	# cases	%		# cases	%		# cases	%	
< 30	5	0.34	-2.84 ± 0.22*	10	1.12	-1.27 ± 0.26	13	0.64	0.77 ± 0.26**
30-39	7	0.48	-2.75 ± 0.21*	14	1.56	-1.16 ± 0.46	218	10.73	0.78 ± 0.22**
40-49	59	4.05	-2.86 ± 1.18*	78	8.71	-1.23 ± 0.51	580	28.56	-0.23 ± 0.51**
50-59	475	32.65	-2.94 ± 0.28*	245	27.34	-1.39 ± 0.47	768	37.81	-0.39 ± 0.47**
60-69	555	38.14	-2.97 ± 0.31*	312	34.82	-1.47 ± 0.52	298	14.67	-0.47 ± 0.52**
> 69	354	24.33	-3.04 ± 0.36*	237	26.45	-1.37 ± 0.61	154	7.89	-0.37 ± 0.21**

*comparison of bone density in age groups in osteoporosis group, ANOVA analysis;

**comparison of bone density in age groups in normal BMD group, ANOVA analysis

**Figure 1:**
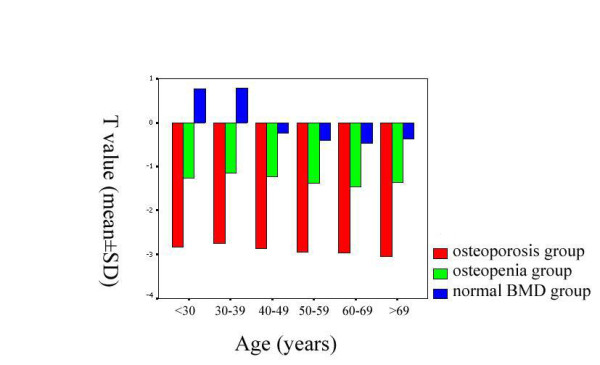
**the trend of T value changes as age increasing of three groups**. Age-stratified analysis showed all of the three groups had the trend that the T value of BMD reached its peak at age 30 to 39 years, started to decline at age 40 to 49 years, and declined significantly from age 60 to 69 years.

Age-stratified analysis showed all of the three groups had the trend that the T value of BMD reached its peak at age 30 to 39 years, started to decline at age 40 to 49 years, and declined significantly from age 60 to 69 years. Analysis revealed that, in the osteopenia and normal BMD groups, bone density decreased with age reaching its lowest level at age 60-69, but later this pattern was not apparent. In the osteoporosis group, among patients > 40 years, bone density decreased as age increased (Figure 1). The percentage of osteoporosis in female patients aged 60-69 years was 38.14%, which took the largest proportion. Bone density of women > 69 years was significantly lower than that of younger patients in all groups, a statistically significant finding by one-way ANOVA test (P < 0.05, Table 2).

### Clinical Symptoms Associated With Op

Table 3 shows the incidence rates of neck pain, shoulder pain, lower back pain, upper back pain, leg pain, leg numbness, arm pain, arm numbness and bone fracture of osteoporosis and non-osteoporosis groups. Both groups had symptoms related to pain and numbness; no significant difference was found in reported upper and lower back pain, or leg pain between two groups (*p *> 0.05). Neck, shoulder and arm pain, leg and arm numbness were more common in the non-osteoporosis group (*p *< 0.05, OR < 1, and upper limit of 95% CI of OR < 1). However, the incidence of bone fracture was significantly higher in osteoporosis group (*p *< 0.05, OR > 1, and lower limit of 95% CI of OR > 1).

**Table 3:** Clinical symptoms in patients with and without osteoporosis.

Clinical symptoms	Osteoporosis (n = 1455)		Non-osteoporosis (n = 2927)		Chi-square value	Odds ratio (OR(95%CI))	P value
				
	# cases	%	# cases	%			
neck pain	639	43.92	1524	52.07	25.823*	0.721(0.635-0.818)^Δ^	0.000
shoulder pain	673	46.25	1613	55.11	30.528*	0.701(0.618-0.795)^Δ^	0.000
lower back pain	1119	76.91	2252	76.94	0.001	0.998(0.860-1.159)	0.981
upper back pain	723	49.69	1445	49.37	0.041	1.013(0.893-1.149)	0.840
leg pain	1003	68.93	2064	70.52	1.157	0.928(0.809-1.064)	0.282
leg numbness	500	34.36	1109	37.89	5.195*	0.858(0.753-0.979)^Δ^	0.023
arm pain	600	41.24	1396	47.69	16.336*	0.770(0.678-0.874)^Δ^	0.000
arm numbness	530	36.43	1196	40.86	8.007*	0.829(0.728-0.944)^Δ^	0.005
bone fracture	254	17.46	120	4.10	222.124*	4.947(3.941-6.210)^Δ^	0.000

*Osteoporosis vs. without osteoporosis, X^2^-test, P < 0.05

^Δ^Logistic regression of relationship between osteoporosis and clinical symptoms

In the osteoporosis group the most common symptoms were lower back pain, leg pain and upper back pain with incidence rates of 76.91%, 68.93%, 49.69% respectively, followed by shoulder pain (46.25%), neck pain (43.92%), arm pain (41.24%), arm numbness (36.43%), and leg numbness (34.36%). In the group without osteoporosis, the most common symptom was lower back pain (76.94%), leg pain (70.52%), shoulder pain (55.11%), neck pain (52.07%), upper back pain (49.37%), arm pain (47.69%), and leg numbness (37.89%).

According to the menstrual status, the patients of osteoporosis were sub-divided into three groups: premenopausal (72 cases), perimenopausal (143 cases) and postmenopausal (1240 cases). Table 4 showed: 1) the maximum proportions of sites of pain or numbness in the three sub-groups were 3 sites (36.11%), 5 sites (30.77%) and 5 sites (30.81%) respectively. The averages of sites of pain or numbness in the three sub-groups were 2.80 sites, 3.50 sites and 3.24 sites respectively, Kruskal-Wallis test manifested that the value of Chi-square was 11.200, p = 0.004, indicating significant differences exited among the three groups. 2) the maximum proportions of duration of the symptoms in the three sub-groups were 6-12 months (20.83%), 36-72 months (20.98%) and >144 months (29.35%) respectively. The averages of duration of the symptoms in the three sub-groups were 36.44 months, 52.95 months and 75.58 months respectively, the value of Chi-square was 17.299, p = 0.000, indicating significant differences exited among the three groups. 3) the occurrences of bone fracture (single + multiple) were 6.95%, 39.16% and 15.57% respectively, the value of Chi-square was 5.067, p = 0.079, indicating there were no significant differences exited among the three groups. (Table 4)

**Table 4:** Sub-group analysis of clinical characteristics related to OP

	Osteoporosis group (n = 1455)		
			
	Premenopausal	Perimenopausal	Postmenopausal
	(n = 72, %)	(n = 143, %)	(n = 1240, %)
**Sites of pain or numbness**			
1 site	14 (19.44)	15 (10.50)	193 (15.56)
2 sites	12 (16.67)	23 (16.08)	262 (21.13)
3 sites	26 (36.11)	25 (17.48)	225 (18.15)
4 sites	14 (19.44)	36 (25.17)	178 (14.35)
5 sites	6 (8.33)	44 (30.77)	382 (30.81)
Average sites	2.80	3.50	3.24
Chi-square	11.200		
P	0.004		
**Duration of the symptoms**			
< 1 month	5 (6.94)	11 (7.69)	59 (4.76)
1-3 months	9 (12.50)	7 (4.90)	49 (3.95)
3-6 months	8 (11.11)	9 (6.29)	58 (4.68)
6-12 months	15 (20.83)	26 (18.18)	72 (5.81)
12-36 months	12 (16.67)	21 (14.69)	257 (20.73)
36-72 months	11 (15.28)	30 (20.98)	131 (10.56)
72-144 months	5 (6.94)	13 (9.09)	250 (20.16)
> 144 months	7 (9.72)	26 (18.18)	364 (29.35)
Average months	36.44	52.95	75.58
Chi-square	17.299		
P	0.000		
**Occurrences of bone fracture**			
Never	67 (93.06)	87 (60.84)	1047 (84.44)
Single	2 (2.78)	42 (29.37)	70 (5.65)
Multiple	3 (4.17)	14 (9.79)	123 (9.92)
Chi-square	5.067		
P	0.079		

### Risk Factors Associated With Op

Data of associated diseases are reported in Table 5. Hypertension, diabetes mellitus and hyperostosis were more frequent in the osteoporosis patients (21.8 vs. 15.1%, X^2 ^= 30.339, p = 0.000; 9.7 vs.5.9%, X^2 ^= 21.320, p = 0.000 and 66.0 vs. 6.4%, X^2 ^= 1786.041, p = 0.000 respectively, P < 0.05). The OR and 95% CI of them were 1.566 (1.334-1.839), 1.719 (1.363-2.168) and 28.342 (23.595-34.045) respectively, which indicated that Hypertension, diabetes mellitus and hyperostosis were the risk factors of osteoporosis.

**Table 5:** Risk factors associated with OP

	Osteoporosis group	Non-Osteoporosis Group	X^2^	p	OR (95%)
	(n = 1455)	(n = 2927)			
**Postmenopausal (n)**	1240 (85.2) *	2159 (73.7)	73.377	0.000	2.052(1.737-2.423)
**Body mass index (kg/m^2^)**	25.6 ± 3.1*	23.4 ± 2.8			
< 20 (n)	25(1.7)	91(3.1)			
20-25 (n)	786(54.0)	1300(44.4)			
≥25 (n)	644(44.3)*	574(19.6)	324.746	0.000	3.441(2.998-3.949)
**Education level (n)**					
Elementary	747(51.3)	1354(46.2)			
High school or equivalent	456(31.3)	898(30.7)			
College or equivalent	252(17.4) *	675(23.1)	19.208	0.000	0.699(0.595-0.821)
**Calcium supplements (n)**	699(48.0) *	1897(64.8)	113.186	0.000	0.502(0.442-0.570)
**Physical activity (n)**					
Never	818(56.2)	1724(58.9)			
Occasionally	405(27.8)	338(11.5)			
Frequently	232(15.9) *	865(29.6)	95.889	0.000	0.452(0.385-0.531)
**Smoking status (n)**					
Current or former smoker	234(16.1) *	142(4.9)	156.280	0.000	3.759(3.019-4.679)
Never smoke	1221(83.9)	2785(95.1)			
**Hypertension (n)**	317(21.8)*	442(15.1)	30.339	0.000	1.566(1.334-1.839)
**Diabetes mellitus (n)**	141(9.7) *	172(5.9)	21.320	0.000	1.719(1.363-2.168)
**Hyperostosis (n)**	961(66.0)*	188(6.4)	1786.041	0.000	28.342(23.595-34.045)

*: compared with non-OP group, significant difference exited.

The most common lifestyle-related risk factors for osteoporosis were smoking, body mass index, lack of physical activity and menopause (Table 5). In osteoporosis group the ratio of current or former smoker was significantly higher than that of non-osteoporosis group (16.1% vs. 4.9%, X^2 ^= 156.280, p = 0.000, OR = 3.759, 95% CI = (3.019-4.679)), which indicated that smoking was a risk factor of osteoporosis. The cases whose body mass index ≥ 25 was more prevalence in osteoporosis group (44.3% vs. 19.6%, X^2 ^= 324.746, p = 0.000, OR = 3.441, 95% CI = (2.998-3.949)), which indicated that the BMI was a risk factor of osteoporosis. In osteoporosis group the ratio of cases who had frequently physical activity was lower than that of non-osteoporosis group (15.9% vs. 29.6%, X^2 ^= 95.889, p = 0.000, OR = 0.452, 95% CI = (0.385-0.531)), which indicated that physical activity was a protective factor of osteoporosis. Regarding menopause status, 1240 cases in osteoporosis group and 2159 cases of non-osteoporosis group were postmenopausal (85.2% vs. 73.7%, X^2 ^= 73.377, p = 0.000, OR = 2.052, 95% CI = (1.737-2.423)), indicating that postmenopausal was the risk factor of osteoporosis. Also, higher educational level and regular calcium supplements were the protective factors of OP (Table 5).

## Discussion

This is the first ten-year longitudinal study with regard to musculoskeletal pain or numbness of patients with osteoporosis in southwest China. We established the OP database to evaluate the clinical features of OP in West China Hospital of Sichuan University, a large hospital with more than 4000 hospital beds. We discuss the novel findings comparing with data from the United States and Europe.

Osteoporosis is predominately found in females. In the European Vertebral Osteoporosis Study of 15,570 men and women age 50-79, [9] osteoporosis affected 3% to 6% of men over 50, whereas another study found the lifetime risk of hip fracture for 50-year-olds in the United Kingdom was 11.4% for women and 3.1% for men [12,13]. The occurrence of osteoporosis is influenced by many factors, especially decreasing rates of peak bone mass and osteopenia after menopause. Current opinion is that lack of estrogen after menopause and aging are the main causes of osteoporosis [14].

Epidemiologic studies indicated the incidence of osteoporosis in females aged 50-59 years is >50% [15] due to changes in endocrine metabolism. Estrogen levels significantly decrease after menopause, especially in the 5-10 years immediately following menopause [16]. Women within three years of menopause who lose bone rapidly are twice as likely to have vertebral and peripheral fractures as their normal or slower-bone-losing counterparts, again similar to BMD predictions [17]. Most cases of osteoporosis occur in postmenopausal women, and incidence increases with age. Bone density is an important determinant of fracture risk, especially in women age ≥ 65 years [18,19]. In the United States, approximately 20% of white women age ≥ 50 years have osteoporosis, defined as femoral BMD > 2.5 SD below the mean of young, healthy white women [5,20]. Another 35-50% have low bone mass, defined as BMD 1-2.5 SD below the mean. Osteoporosis rates vary with ethnicity, with the highest rates in whites and those of Asian descent and the lowest rates in blacks [21]. Our present study also showed that the incidence of osteoporosis increased after the age of 50 years, reaching its peak in patients aged 60-69 years. Therefore prevention strategies should be aimed at this cohort.

Interestingly, we found that musculoskeletal pain and numbness are prevalent clinical manifestations among hospitalised patients on Rehabilitation medicine wards. Sometimes this group of patients was misdiagnosed as osteoporosis. As we know, musculoskeletal pain and numbness may be one of common symptoms of osteoporosis, followed by shortness, dowager's hump, bone fracture and respiratory disorder. Previous data showed 67% of osteoporosis patients had localized lower back pain, 9% had lower back pain and extremity radiating pain, 10% had lower back and girdle pain, 4% had lower back pain and numbness, 10% had lower back pain, limb numbness and numbness/weakness in the lumber [22].

In this study, our data showed there was no correlation between shoulder pain and arm pain and osteoporosis, thus shoulder pain and arm pain alone could not support the diagnosis of osteoporosis. Part of the explanation may be pain was most common symptom of osteoarthritis, rheumatoid arthritis, or other degenerative joint diseases. Consequently, lower back pain, leg pain and numbness had no relative specificity to a diagnosis of osteoporosis, and differential diagnosis is needed to distinguish osteoporosis from a slipped disc or muscle strain. Our results showed that musculoskeletal pain and numbness had no significant correlation with osteoporosis, indicating the need for differential diagnosis to exclude osteoporosis. In addition, severity of pain or numbness and no correlation with bone density and number of symptoms did not indicate osteoporosis severity. While the occurrence of osteoporosis is positively correlated with symptoms bone fracture, indicating that bone fracture has the value in diagnosing osteoporosis. Further, our study showed no correlation between disease duration and bone density. Bone density was relatively low among patients only 3-6 months into the disease. Disease course and patient age should be considered together when evaluating osteoporosis severity.

Many studies have shown that better-educated individuals tend to exercise more, smoke less, and have better maintenance of body weight. Better educated individuals also tend to adopt healthier eating habits, including more dietary calcium, vegetables, soy foods, and fruits and less saturated fat and alcohol [23-28]. An association between higher intakes of calcium, soy, fresh fruits or vegetables with better bone mass in postmenopausal women has been reported [25-29]. Such education-associated favorable dietary habits might play a role in the improvement of bone health. In univariate analysis, our results have demonstrated that that increases in educational level are associated with lower risks for osteoporosis (P > 0.05). It reminds us that we should pay more attention to the patients' educational status in many areas of developing countries.

Clinical data had shown osteoporosis as the main cause of pathologic fracture in older adults, often leading to disability and death. The morbidities, distribution and factors associated with osteoporosis vary by country and region. The ability to understand its risk factors, at-risk populations and identification before bone fracture can greatly aid in osteoporosis prevention and treatment. Traditional epidemiologic studies of osteoporosis include analysis of bone fracture incidence and prevalence, while clinical methods include measuring bone mass or BMD.

The known risk factor is a high-risk population with no bone fracture but decreased bone mass or BMD. In general, lower BMD scores indicate more severe osteoporosis and higher risk of fracture. A decrease of 1 SD in BMD represents a 10% to 12% decrease in BMD and an increase in fracture risk of 1.5 to 2.6 [30,31]. BMD and fracture risk are most closely related when bone density is used to predict fracture risk at that site. In our study, the results from multiple regression analysis indicate that associated complications including hypertension, diabetes mellitus and hyperostosis are as important risk factors of low BMD as the lack of physical activity, loss of height and smoking. This indicates that the treatment guidelines should be established in this region according to risk-related factors.

The development of the FRAX^® ^models for fracture risk assessment has been based on a programme of work undertaken at the WHO Collaborating Centre for Metabolic Bone Diseases at Sheffield University. Clinical risk factors included age, sex, weight, height, previous fracture, parent fractured hip, current smoking, glucocorticoids, rheumatoid arthritis, secondary osteoporosis, alcohol 3 or more units/day, and Bone mineral density (BMD). Our study excluded secondary (i. e. steroids, plasmocytoma, or renal) osteoporosis and focused on primary osteoporosis. We expected to combine China's national conditions compared with the international level using ten years data to guide the diagnosis and treatment for OP in worldwide primary hospital.

Furthermore, this study has showed different conclusion from previous study with regard to BMI and osteoporosis [32-34]. It has been established that BMI is a protective factor against osteoporosis, and it is also included as protective factor in FRAX [35-37]. Based on the research of this database, we considered BMI was not a protective factor for OP. Therefore, we infer that the difference of ethnicity may affect the effect of obesity to female osteoporosis. The updated study reported that BMD at the spine and hip have significant genetic determination, BMI is more likely to be affected by environmental factors than BMD [38]. In addition, BMD at the spine and hip shares more genetic effect (pleiotropy) than BMI and BMD do in Chinese Han ethnicity, though the effects are significant for both [38]. Previous study found that the contribution of ADRB3 genotypes to the prediction of BMI, BMD and fracture risk is limited in Caucasian population [39]. Thus, several shared genomic regions for BFM and BMD were identified and may benefit further positional and functional studies, aimed at eventually uncovering the complex mechanism underlying the shared genetic determination of obesity and osteoporosis [40]. As for Asian anthodium female, high BMI is not a protective factor for bone mineral density (BMD), inversely, can be a risk factor. This finding was consistent with previously published work. Further studies are necessary to clarify this hypothesis.

## Conclusions

To the best of our knowledge, the present study offers the first reference data of the relationship between epidemiologic distribution of osteoporosis and associated factors in adults Chinese women. These reference data will assist in diagnosis of osteoporosis by DEXA in Chinese women. DEXA testing is a critical step to appropriate osteoporosis treatment and fracture prevention. As discussed, better understanding of the clinical characteristics of patients may lead to improved management and reduced risk of fracture. Larger investigations are necessary to understand the prevention and cure strategies most appropriate for Chinese females. It calls for vigorous measures to implement public health education and professional training to provide sustainable long-term medical services within the existing health care infrastructures in this region. The present study has several limitations. We acknowledge this retrospective study was designed ten years ago and should advance with the times. Meanwhile, we recognise that a large prospective cohort study would be required to verify the current findings.

## Competing interests

The authors declare that they have no competing interests.

## Authors' contributions

CH conceived of the study, and participated in its design and coordination and drafted the manuscript. SL participated in the design of the study, performed the statistical analysis, and polished the manuscript. MD carried out investigative studies, participated in the designed the database forms and drafted the manuscript. HH participated in the design of the study and performed the statistical analysis. All authors read and approved the final manuscript.

## Pre-publication history

The pre-publication history for this paper can be accessed here:

http://www.biomedcentral.com/1471-2474/11/183/prepub
